# Fourth Wes Fabb Oration Diversity of primary care in Asia Pacific: pathways to convergence

**DOI:** 10.1186/1447-056X-9-7

**Published:** 2010-03-26

**Authors:** Zorayda E Leopando

**Affiliations:** 1 Department of Family and Community Medicine, University of the Philippines Manila, Manila, Philippines

## Abstract

Asia Pacific is one of the 6 regions of the World Organization of Family Doctors (Wonca). It is a region with 16 full and 2 associate members coming from 14 countries. One of the main activities in the region is the regional conference highlighted by the Wes Fabb Oration.

This Fourth Wes Fabb Oration has a historical perspective and three main parts: the results of a cross sectional survey done among member organizations and three countries not affiliated yet with Wonca which show Family Medicine as to status, practice, education and research; the regional initiatives and activities which indicate convergence; and, suggested action points which can further promote family medicine development, collaboration, and convergence.

## Introduction

One of the great events in the World Organization of Family Medicine (Wonca) Asia Pacific Region is the establishment of Professor Wes Fabb Oration in 2001, an initiative done to honor Wes and his contribution to the development and strengthening of family medicine around the world. Wes served Wonca for more than a quarter of a century, first as honorary secretary and at the time of his retirement in 2001, he was chief executive officer. The Wes Fabb Oration also honors Asian family physicians who have personified what Wes stands for and who have similarly contributed to family medicine growth and the improvement in health and well being of patients.

It is a great honor to deliver the Fourth Wes Fabb Oration in the birthplace of Professor Fabb and Wonca, after three previous orators who are giants of family medicine - Professor Fabb himself, and former Wonca world presidents Dr. Peter CY Lee and Dr. MK Rajakumar. I thank the Royal Australian College of General Practitioners and Wonca Asia Pacific Region for this honor.

I had the privilege of working closely with Professor Fabb, whom I consider to be one of my mentors. His love for family medicine and Wonca is a lifelong commitment, similar to his belief in lifelong medical education. Thus, being webmaster of Global Family Doctor after his retirement as chief executive officer and establishing the *Journal Alert *was not a surprise. Retiring as webmaster, he continued as one of its medical editors.

Professor Fabb's guiding principle is that "*Wonca is inclusive," *a principle he infected us with, and is contributory to the growth of Wonca. He is consistent with his belief in what family medicine and Wonca stand for. Reading through articles he has written in more than 30 years, what stand out are his advocacies for optimum quality and comprehensive care in family medicine and high standard of family medicine education [1-3].

In what way did Professor Fabb influence my career and advocacies in family medicine? As regional president (then called regional vice president) for Asia Pacific, reaching out to member organizations through personal visits and constant communications at the time when the internet is not that popular yet, I would say, brought the member organizations closer to Wonca. In addition, we were able to have our own structure as a region, with its own set of regional executives and bylaws like Europe. Member organizations were encouraged to join forces with governments and medical schools bringing with them the *"Core curriculum for family medicine residency*" and the London Ontario proceedings on *"Making health system more relevant to people's needs: the contribution of family doctors*." Strengths of each member organization and needs of others were matched to facilitate collaboration. Workshops were institutionalized in the promotion of high standards of family medicine education, research workshops and contests were held and the foundation for *Asia Pacific Family Medicine*, the official scientific publication of Wonca Asia Pacific, was laid down. Thus, by 2001 when my term as regional president ended, we had scheduled regional conferences up to 2008, called for submission of papers to *Asia Pacific Family Medicine*, instituted the journal's peer review process, and approved the Wes Fabb Oration.

The inspiration from Wes and the theme of the conference motivated me to choose the topic **"Diversity of primary care in Asia Pacific: pathways to convergence" **for this Fourth Wes Fabb Oration.

## Historical perspective

Asia Pacific is one of the 6 regions of the World Organization of Family Doctors (Wonca). Serving 2.07 billion people, it is the only region that extends from the northern to southern hemispheres. Countries in the region are so diverse in geographic size, population, religion, economics, politics, culture, and health care.

**Figure 1:**
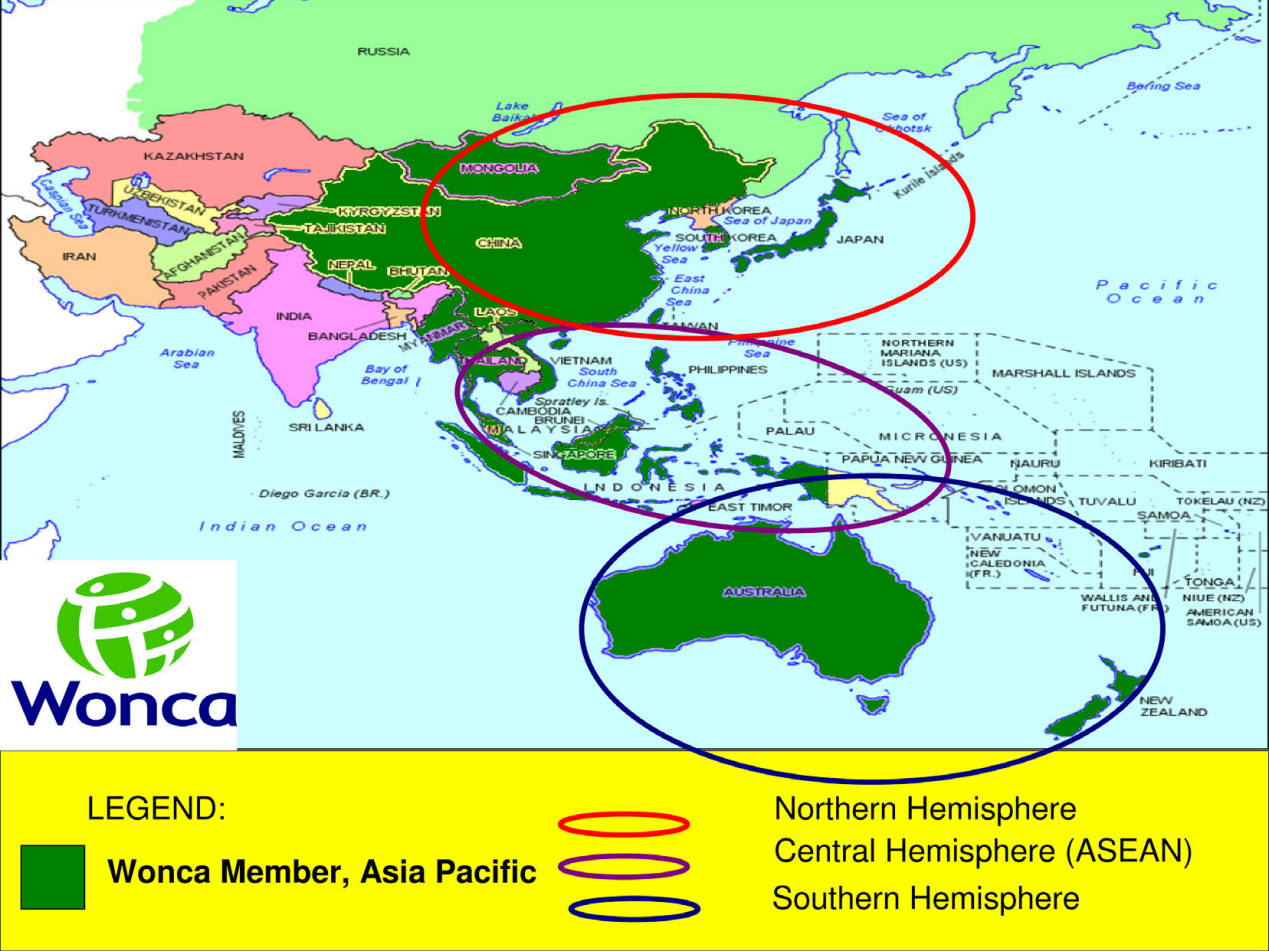
**Map of Wonca Asia Pacific Region**.

If we group the member organizations according to hemisphere of location, northern hemisphere will have organizations from China, Hongkong, Japan, Macau, Mongolia, South Korea, and Taiwan. Central hemisphere will be the ASEAN region currently with organizations from Indonesia Malaysia, Myanmar, the Philippines, Singapore, Thailand and Vietnam. Southern hemisphere has organizations from Australia, Fiji and New Zealand. (See Figure 1)

**Figure 2:**
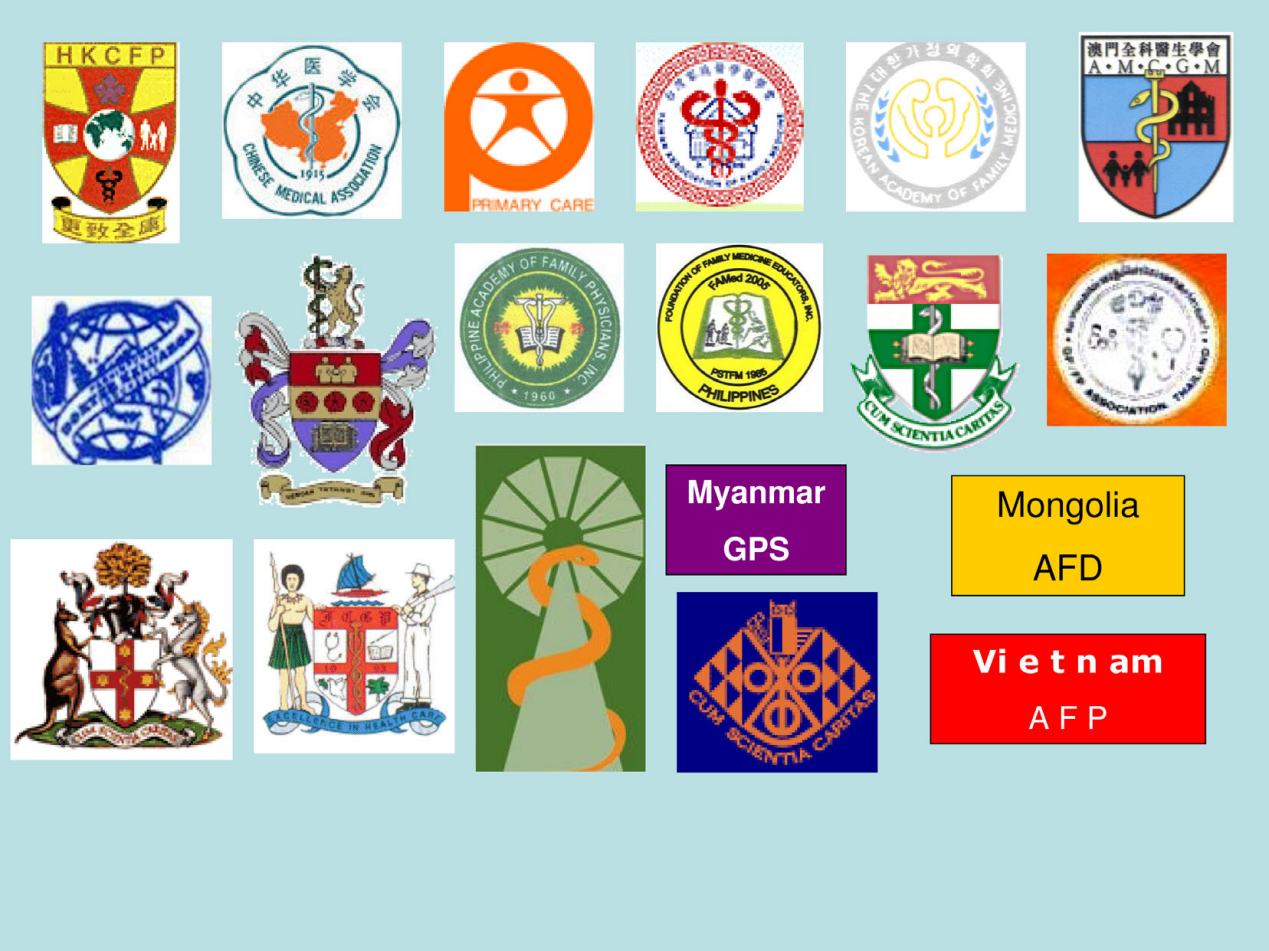
**Member organizations of Wonca Asia Pacific Region**.

Wonca Asia Pacific has 16 full and 2 associate members. (See Figure 2)

Primary care in Asia Pacific is anchored on family medicine and general practice. The training and practice of primary care is diverse due to varying influences from the United Kingdom and United States of America.

Established in 1972, the Wonca has worked tediously in hastening the development of Family Medicine. Through the efforts of its members, Wonca was able to conduct educational workshops which formulated the core curriculum for family medicine, as well as the core content of examination. Research workshops gave way to the publication of several journals, including the Asia Pacific Family Medicine [4,5]. In addition, Wonca, both globally and regionally, has undertaken activities that guide countries in developing, strengthening, and enhancing primary care. Global activities include the 1994 Wonca - WHO strategic meeting in Canada, Global Family Doctor website, 2002 Guidebook, and 2003 Research Workshop. Among the regional activities in Asia Pacific were regional conferences, regional structure and bylaws, the struggling *Asia Pacific Family Medicine *journal and family medicine education workshops.

WONCA has worked closely with the World Health Organization (WHO), which in 1978 identified primary health care approach as the strategy to attain the global target of "Health for All by the Year 2000." With family physicians and general practitioners at the forefront of care, Wonca supported the initiative. In the WHO-Wonca conference on contribution of family doctors, the executive summary was very challenging and still very relevant and I quote: "*to meet people's needs, fundamental changes must occur in the health system, in the medical professions, and in medical schools and other educational institutions. The family doctor should have a central role in the achievement of quality, cost-effectiveness, and equity in health systems. To fulfill this responsibility, the family doctor must be highly competent in patient care and must integrate individual and community health care *[6]." I am thankful that when I became Regional President for Asia Pacific, I had the London Ontario document with its 14 vision statements on optimal medical care, optimal medical practice, and optimal medical education; and 21 specific recommendations which set the direction on how I would function in my office and in how I would lead the member organizations toward the Wonca goal of excellence and relevance in family medicine practice and education. The action points derived from the 1994 joint Wonca - WHO strategic forum on the "Contribution of the family doctor," were monitored in the Asia Pacific Region from 1995 to 2001. Twinning between organizations was encouraged, and more frequent regional conferences were held, thus bringing member organizations closer to Wonca and with each other [7-9]. WHO's focus on family medicine was further intensified in the late 1990s through the "Towards unity for health" campaign, which integrated individual health with population health and clinical medicine with public health.

## Diversity in status of primary care and family medicine development

An internet cross-sectional survey of all member organizations in Asia Pacific and non member organizations in countries like Brunei, Laos and Cambodia was done to determine the status of family medicine development. There was a response rate of 86.4%. Respondents were distributed as follows: 6 from northern hemisphere, 10 from central hemisphere and 3 from southern hemisphere.

The 19 respondents included national office bearers, Wonca council members, and academic department heads and three respondents from organizations not yet affiliated with WONCA. Table 1 shows summary of results. (See Table 1)

**Table 1:** Specialty Recognition of Family Medicine in Asia Pacific, 2006-2008.

Organization Giving Recognition	HEMISPHERE			TOTAL
	
	Northern	Central	Southern	
National Medical Organization	4	6	3	13

Policy making body of Medical Education	5	6	2	13

Department of Health/Ministry of Health	2	5	3	10

General Medical Council	3	3	3	9

National Health Insurance	3	2	2	7

(n = 19)

### 1. Specialty status

Family Medicine is recognized as a specialty according to 13 respondents and recognition was given mainly by the national medical organizations, policy makers on medical education, and the departments of health. (See Table 1) In the 1960s, Professor Ian Mc Whinney, explaining why family medicine is a specialty, emphasized its distinct core knowledge, unique field of action, training which is intellectually vigorous, and active area of research [10].

### 2. Practice of Primary Care

On the role of family physicians in health care, 52.5% of respondents revealed the absence of law or policy mandating family physicians to be the entry point of health care and only 37% affirmed its presence. All 7 with mandate reported finishing a medical degree with license to practice as requirement. Only 3 identified board qualified and/or trained family physicians as prerequisite to practice. Of the 10 with no mandate, 8 have no plan to work for a policy. But do we need a policy? Barbara Starfield in her studies showed that countries giving greater emphasis on primary care spend less on health care and have better outcomes. Primary care physicians provide first contact, comprehensive care and entry into the health delivery system is one of the characteristics [11].

Multiple answers on locus of care showed that family physicians provide continuity of care geographically. All answered community-based clinic followed by home care. More than 50% answered hospital-based clinic/surgery and industrial/occupational clinic. These are considerations in training family physicians because different areas have unique needs.

The predominant level of care provided is primary care at the outpatient level, sometimes with inpatient component or at secondary level of care. Family physicians have mixed sector affiliations working with both the government health centers and the private sector for a fee. Less than half are with national health insurance or private health maintenance organizations (HMOs). 52.6% answered no patient registration while 36.8% mentioned that patient may either be listed with a family physician or a health facility. About 52% of respondents indicated the presence of formal referral policy and mechanism while 36.8% have none.

In the 1990s, most countries underwent health sector reforms, which included health financing through social insurance. More than half answered selective health insurance scheme, while a third answered that there is a national health service with universal coverage. Another third said there is free public sector at health center and fee for service private sector, indicating combination of ways by which health care is financed. Professor Goh Lee Gan mentioned in an article that improvement of payment scheme for primary care physicians is an important issue for survival [12].

### 3. Academic development

Around 68% of organizations confer academic recognition to members. However, terminologies for ranking are varied with the title of fellow as more commonly used. Majority (14/19) have certifying specialty examination, and another majority (13/19) have no system of recertification.

Compared to the 1992 survey on academic programs, the recent survey revealed that there are more countries offering undergraduate program in family medicine. Presently, there are 16 countries offering residency or vocational training. There were only 10 in 1992[13]. Also available are continuing professional development and/or graduate programs. (See Table 2)

**Table 2:** Available Family Medicine Academic Program/s in Asia Pacific, 2006-2008

Training Program	HEMISPHERE			TOTAL
		
	Northern	Central	Southern	
Undergraduate Program (Medical curriculum)	5	9	2	16

Residency/Vocational training	6	8	2	16

Continuing Professional Development Program	5	5	3	13

Graduate degree (Master)	4	6	2	12

Diploma Course	3	5	1	9

(n = 19)

When asked to characterize family medicine undergraduate educational program, it is noted that:

• Fourteen have family medicine course, thirteen have clinic rotation and Family Medicine is offered in all medical schools in eleven.

• Ten offer Family Medicine with Community Medicine but is offered as solo subject in 9.

• Eight respond that Family Medicine integrates clinical medicine, behavioral science and public health.

Sixteen reported having residency/vocational training in Family Medicine. Learning objectives include expected competencies and characteristics of family physicians (68.8%), preparation for practice of family medicine (37.5%), and domain (3/19).

The guiding principles used in selecting the learning strategies are: adult learning, problem-based learning, and learning by doing. Strategies include learning with patients such as: hospital and practice postings, bedside rounds, direct observation or review of video tapes of consultation. Learning with supervising physicians during conferences entails: case discussion/presentation, lectures and workshops done either as monthly intensive courses, modules, or weekend sessions. Self-directed learning includes learning portfolio and its review, distance learning, and learning contracts.

Importance is also given to evidence-based medicine, quality assurance and research, with chart review, medical audit, critical appraisal of the literature, mortality and morbidity review.

The venue for clinical training is mixed hospital and practice based clinic in the community.

The most popular rotations for the residency training are: 94% each for Internal Medicine, Pediatrics, and Surgery, which are higher than the combined Family Medicine/Primary Care/Health Center rotations. Considering that we are in family medicine, we would expect 100% inclusion. Other rotations given by more than 50% of respondents are: Obstetrics and Gynecology, Mental Health and Psychiatry, and Emergency Medicine.

Evaluation of trainees is both formative and summative. Feedback of the evaluation is an important feature. Formative assessment covers observational rating scale for marking, review of portfolio and processing of video tapes. Summative evaluation is done through written and practical examinations which use multiple choice questions, objective structured clinical examinations (OSCEs) and long cases. In addition, graded case report and thesis are reported. Evaluation of training programs is done through accreditation and trainees' feedback evaluation of their experiences.

Program accreditation is done by the Ministry of Health at the national or provincial level, certifying board, and the national accrediting committee. Accreditation is conducted every 1 - 6 years using set criteria and standards.

### 3. Research and quality activities

Quality activities are important to family medicine. Professor Fabb has promoted this since 1990, and Wonca has a world committee for this. Of the 9 respondents answering this question, 8 identified the following activities: practice standards in family medicine; quality activities such as evidence-based medicine, utilization of clinical practice guidelines and medical audit. Six mentioned that physicians are required to participate in quality activities with 4 reporting actual engagement in quality projects. Mechanism of practice review through peers/experts visit for assessment based on standards is mandatory in 3 but optional in 2.

Research is an important component of family medicine as a specialty. Activities for research capability building include research writing workshop, research proposal making workshop, and research consultants assisting members. Other research-related activities have something to do with research funding, technical and institutional review, and practice based research network.

Research dissemination activities include research presentation in annual scientific assembly topping the list followed by submission of abstracts to Wonca conferences and research contests.

Nine reported that papers are submitted to *Asia Pacific Family Medicine*. Only 9 each have their own peer reviewed journal or give out awards to outstanding researchers.

### 4. Best Practices and needs lead to twinning opportunities (See Table 3)

Table 3 shows twinning opportunities of areas with best practices with significant problems and needs for assistance. The best practices make it easier for Asia Pacific to establish a database on resources available. Thirteen reported on their best practices, which included community based practice or community oriented primary care; RACGP conjoint examination; training programs, including teacher training; on line teaching; and organized tutors group. Significant problems and frustrations cited by 14 respondents include: recognition/antagonism, lack of funds, member participation, role of family physicians in health care, and migration. Needs for assistance expressed were on research issues, networking, improvement of training programs, trained teachers, collaboration with Wonca, legislation on family physician's role, and regional recognition of programs.

**Table 3:** Twinning Opportunities, Asia Pacific Region

Best Practices	Significant Problems	Needs for Possible Assistance
• Community--based practice/community oriented primary care • Conjoint examination • Training programs • On-line teaching • Availability of consultants • Organized Teachers Group • Primary Care policy of the government	• Recognition/Status/Prestige • Lack of funds • Antagonism • Member participation • Role of family physicians in health care • Website not sustained • Migration	• Research issues • Networking • Improvement of training programs • Trained teachers • Collaboration with Wonca • Legislation on Family Physician's role • Regional recognition of programs

### Pathways to convergence

The pathways to convergence in the midst of diversity, as indicated by the results of the above cited survey, start with what we already have and what we in Asia Pacific can tread on.

***1. WONCA 2007 Resolutions ***on "*Support to Hamilton Equity Recommendations (HER) Statement and gender equity*", "*A family physician for every family*" and "*Family Medicine taught in all medical schools*" are good starting points to review what the member organizations can collectively advocate. Some have started and experiences can be shared.

***2. Asia Pacific Family Medicine***, our constant forum for convergence and sharing. Support for the journal can be shown through submission of more articles, writing letters to editors on what to improve and nominating peer reviewers.

***3. Primary care research network ***has started but participation in face-to-face meetings and virtual discussions has not included all member organizations. To start collaboration, the region has chosen "Family life cycle in Asia Pacific," "Chronic disease management strategies" and "Teaching related research" as the priority research agenda.

***4. Family Medicine education workshops ***have been done in conjunction with most regional conferences. During the Training of Trainors (TOTs) workshop held in Vietnam, it was agreed that TOTs will be held in areas where they are most needed. This is easy because there are available experts in the region waiting to be tapped.

5. There is a clamor for a ***common regional examination***. At present, three countries have conjoint examination with RACGP. Will RACGP examination be the regional examination or will it be the gold standard?

***6. Twinning ***is happening between Singapore and Myanmar, Thailand and Cambodia, Hongkong and China, the Philippines and Vietnam, Australia and Fiji, and Korea and Mongolia.

***7. WONCA Rural Health ***emanated from Australia, and its conferences have been held in China, Malaysia, and Melbourne. There is a need to encourage rural doctors to be active in the Working Party. Many counties are not yet integrated into the party. In addition, we need to ensure that rural health stream will be included in all regional and world conferences.

***8. WONCA Working Party for Women and Family Medicine ***has championed Gender Equity with HER Statements and 10 Point Ways. Three sessions are included in this conference, but there is a need to ensure that sessions on women are sustained. More important to anticipate is what each member organization will be doing to promote gender equity in between regional and world conferences.

***9. ASEAN region primary care***. In one of the issues of *Asia Pacific Family Medicine*, Professor Richard Hays mentioned the need for the central hemisphere to develop family medicine not only in practice but also in education [14]. The central hemisphere or ASEAN has 10 countries with economic and cultural links. A political partnership through a constitution similar to EU is rapidly gaining ground. Mutual recognition agreements have been signed for various professions. ASEAN region primary care (ARPAC) conference has been held and plans for collaboration on many issues are forthcoming.

## Additional action points

1. ***Revitalization of Regional Committees ***on Classification, Quality, and Informatics with region wide activities. Database on consultants, useful resources, researches, and family medicine development projects are needed. The Regional Executive and Council members should ensure a favorable environment to further push projects such as Classification, Quality and Informatics across member organizations.

2. ***Developments ***include expanding the involvement of women and young family physicians. Young women have met in Singapore and will meet here in Melbourne to address various issues and concerns and how to do so. Young men have issues and concerns too. The Rajakumar Movement will be launched here in Melbourne. This is the start of collaboration among registrars/residents and other trainees in Family Medicine. The women and the youth are clamoring for improved training conditions and smoother transition from training to practice. This can be possible if Wonca Asia Pacific can ensure that countries can lobby for stronger primary care, grant specialty status to Family Medicine, guarantee that all families will have family physicians. Perhaps, strengthening relations with regional offices of WHO can be a key element in this regard.

3. **New activities which may be introduced **are the formation of Asia Pacific Family Medicine Network of Academic Departments which can champion not only resource sharing and faculty and student exchanges but also a core training program for family medicine with heightened learning experience in community-based family medicine because this best simulate the practice. The time is ripe for the conduct of a Regional Workshop on definition of Family Medicine and role of family physicians in health care. Last but not least, a Regional standards and International accreditation of residency/vocational training programs are necessary because we are seeing health migrations not only of patients but family physicians.

## Conclusion

The journey to convergence in the midst of diversity started many years ago. We have most of the pieces in place. We have dedicated advocates of family medicine who are willing to work and help. We have family medicine statesmen in our region led by Professor Wes Fabb who continues to inspire and provide us with guiding light. The pathways have been paved through the cumulative efforts of Wonca leaders in the region. With many of us already raring to make giant strides, there remains only one option- that is making Professor Fabb's dream for family medicine excellence for Wonca Asia Pacific come true.

## Competing interests

The author declares that she has no competing interests.
